# Heterologous Overexpression and Mutagenesis of the Human Bile Salt Export Pump (ABCB11) Using DREAM (Directed REcombination-Assisted Mutagenesis)

**DOI:** 10.1371/journal.pone.0020562

**Published:** 2011-05-31

**Authors:** Jan Stindt, Philipp Ellinger, Claudia Stross, Verena Keitel, Dieter Häussinger, Sander H. J. Smits, Ralf Kubitz, Lutz Schmitt

**Affiliations:** 1 Institute of Biochemistry, Heinrich-Heine-University, Düsseldorf, Germany; 2 Clinic for Gastroenterology, Hepatology and Infectiology, Heinrich-Heine-University, Düsseldorf, Germany; University of Cambridge, United Kingdom

## Abstract

Homologous recombination in *Saccharomyces cerevisiae* is a well-studied process. Here, we describe a yeast-recombination-based approach to construct and mutate plasmids containing the cDNA of the human bile salt export pump (BSEP) that has been shown to be unstable in *E. coli*. Using this approach, we constructed the necessary plasmids for a heterologous overexpression of BSEP in the yeast *Pichia pastoris*. We then applied a new site-directed mutagenesis method, DREAM (Directed REcombination-Assisted Mutagenesis) that completely bypasses *E. coli* by using *S. cerevisiae* as the plasmid host with high mutagenesis efficiency. Finally, we show how to apply this strategy to unstable non-yeast plasmids by rapidly turning an existing mammalian *BSEP* expression construct into a *S. cerevisiae*-compatible plasmid and analyzing the impact of a *BSEP* mutation in several mammalian cell lines.

## Introduction

Recombinant protein expression is a frequent necessity for biochemical studies of proteins, which cannot be obtained in high amounts from their natural source. Among these are many membrane proteins. Extensive expression screening is a vital initial step in the study of membrane proteins, and this stage involves substantial work with recombinant DNA to create the necessary expression constructs. Standard techniques of molecular biology, however, become limiting when working with gene sequences that are unstable in *Escherichia coli*. This is especially encountered in the case of mammalian membrane proteins [1], [2], [3]. Comprehensive studies that address the actual cloning, propagation, and manipulation of constructs containing these unstable DNA sequences are rare and often lack detailed descriptions of the difficulties and, more importantly, of the solutions. In the case of the human bile salt export pump (BSEP, *ABCB11*), the cloning of the cDNA into an expression vector only succeeded after a tremendous amount of work [2]. This process resulted in a plasmid with several point mutations in the coding sequence, six of which changed the sequence on the protein level. A general issue encountered during propagation and targeted mutagenesis of *BSEP*-containing plasmids is the loss of various parts of the *BSEP* cDNA sequence in *E. coli* [2]. This loss of sequences has been observed for other proteins as well [4]. The corresponding DNA sequences have generally been termed “unstable” or even “toxic”, because the presence of the intact plasmid ultimately resulted in bacterial cell death.

One approach to create plasmids containing these “toxic” DNA fragments is to assemble it by homologous recombination in *Saccharomyces cerevisiae (S. cerevisiae)* thereby circumventing *E. coli* [5], [6], [7]. *S. cerevisiae* is able to recombine several overlapping fragments into one circular plasmid containing the desired cDNA. By incorporation of a suitable origin of replication (Ori) as well as a selection marker virtually any plasmid can be created for usage of recombination-based cloning by *S. cerevisiae*. A fragment containing both ORI and selection marker can be added together with the toxic target cDNA in a single recombination step yielding an intact and most importantly stable expression plasmid.

We have used *S. cerevisiae* to create such an expression plasmid containing the “toxic” coding sequence of human BSEP which was subsequently used for BSEP expression in *S. cerevisiae* and *Pichia pastoris (P. pastoris)*. Severe hereditary diseases of the liver are directly associated with mutations in the BSEP transporter [8], [9]. Previously heterologous BSEP expression was only demonstrated in insect cells [2], which hampered a detailed analysis of function of both wild-type BSEP and clinically relevant mutations *in vitro*. Besides creating an expression plasmid for BSEP in *P. pastoris* the method described here is also used to directly create BSEP mutants in the yeast plasmid for subsequent expression in mammalian cell lines. This highlights the applicability of this method to both “simple” expression systems like the yeast based as well as more sophisticated expression in mammalian cell lines.

## Results

### Cloning and expression of BSEP

**Figure 1:**
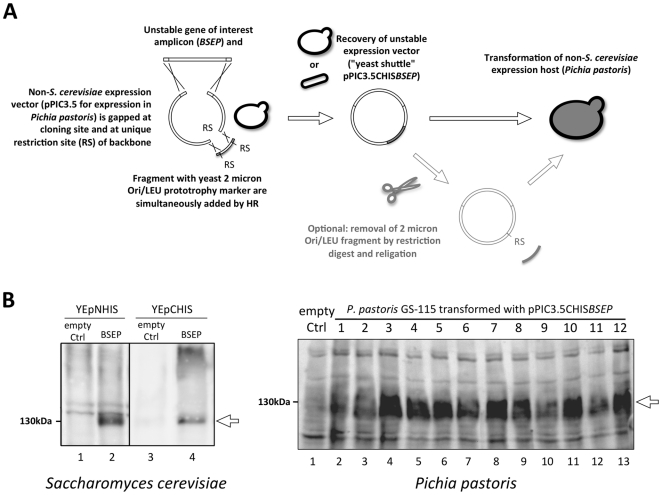
Heterologous overexpression of BSEP in *Saccharomyces cerevisiae* and *Pichia pastoris*. **A,** Toxic or unstable expression plasmids can be constructed for any system in *S. cerevisiae* by adding the necessary sequence to the plasmid backbone. In order to clone BSEP into the *Pichia pastoris* expression cassette on pPIC3.5, the recombination vector was double-digested to allow the simultaneous insertion of both the unstable *BSEP* coding sequence and a PCR-generated fragment of the YEpHIS plasmid carrying the 2 micron origin (Ori) of replication and the leucine (LEU) prototrophy marker by homologous recombination (RS = *Nde*I). This plasmid was recovered from *S. cerevisiae* and obtained in preparative amounts from *E. coli* by strict cultivation at 30°C under suitable conditions. **B,** Expression of human BSEP in *S. cerevisiae* and *P. pastoris*. Equal amounts of whole yeast cell extracts were resolved on SDS-PAGE, electroblotted and probed with the polyclonal BSEP antiserum K168. **Left panel,** Homologous recombination was used to construct both *BSEP* expression vectors directly in *S. cerevisiae*. NHIS/CHIS, N- or C-terminal his tag position; empty Ctrl, strain transformed with corresponding empty YEpHIS expression plasmid. **Right panel,** pPIC3.5-*CHISBSEP* was constructed as described in A and used to transform *P. pastoris* strain GS-115 by electroporation. Empty Ctrl, *P. pastoris* GS-115 strain transformed with the empty pPIC3.5 integration vector.

The unicelluar eukaryote *S. cerevisiae* was initially chosen because of three advantages: (i) it can perform efficient homologous recombination [5], [7]; (ii) expression of other eukaryotic ABC transporters has been successfully reported [10]. For example, *S. cerevisiae* has been used to express the BSEP homologue MDR1 [11], [12]. (iii) Transformants resulting from *in vivo* homologous recombination can immediately be tested for target protein expression. We used these advantages for BSEP, but expression levels in *S. cerevisiae* were very low and not sufficient for subsequent purification or activity studies (Figure 1B, left panel).

Therefore, we changed the expression system from *S. cerevisiae* to *P. pastoris*. Here, expression is driven from the strong inducible *AOX1* promoter. In addition, this yeast strain can reach high cell densities and thereby lead to substantial amounts of membrane protein [13], [14], [15]. Furthermore, Chloupkova et al. were able to express 25 human ABC transporters in *P. pastoris* [15], however BSEP was not among them. We used the *P. pastoris* integration vector pPIC3.5, which was prepared for manipulation in *S. cerevisiae* by integrating the relevant sequence that is necessary for maintenance (ORI) and selection in this yeast. A PCR product containing the *S. cerevisiae* 2 micron ORI and a leucine prototrophy marker and a second PCR product containing *BSEP* with an C-terminal his_8_ tag (kind gift of Dr. Kenneth Linton) were simultaneously recombined into pPIC3.5 *in vivo* in *S. cerevisiae* (Figure 1A). The resulting derivative pPIC3.5-C_his_ *BSEP* (Figure S1) is identical to the construct that would be obtained by conventional bacterial cloning, with the exception of the introduced ORI and selection marker. The plasmid was used to transform *P. pastoris*. Abundant colonies were obtained and twelve of these were subsequently analyzed for BSEP expression. As demonstrated by Western blot analysis, all of the tested clones expressed BSEP at similar levels (Figure 1B, right panel). A strain transformed with the empty integration vector was used as a negative control. The yield of recombinant BSEP achieved in *P. pastoris* is substantially higher than in *S. cerevisiae* allowing further purification and subsequent biochemical analysis.

### DREAM - A site-directed mutagenesis method for unstable and toxic plasmids

Several severe hereditary diseases are known to be associated with human ABC transporter genes [16]. To date, 146 BSEP mutations have been reported in the Human Gene Mutation Database [17], The vast majority of which are associated with liver diseases. One of the most frequently used methods to generate specific mutations is the site-directed mutagenesis (SDM) procedure [18]. This method relies on the usage of *E. coli* to turn the linear product obtained by an *in vitro* mutagenesis into a circular plasmid via nick repair. However, since the cDNA BSEP is toxic for *E.coli*, this standard method is not applicable. Therefore, we used *S. cerevisiae* as host.

**Figure 2:**
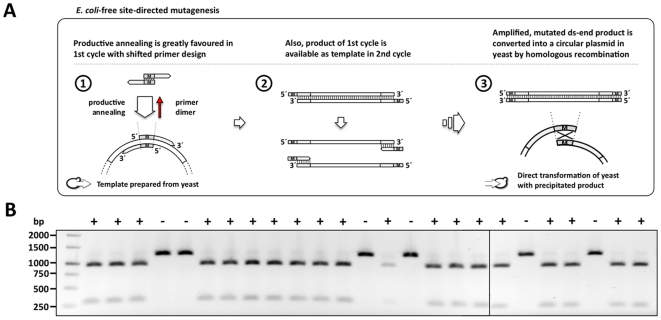
The DREAM method allows site-directed mutagenesis of an unstable *BSEP*-carrying plasmid without the need for *E. coli*. **A,** The product yield can be increased significantly by shifting the primer binding sites from a complete to a partial overlap that allows binding of the primers to product in subsequent cycles. The minute amount (10 ng) of non-mutated template used in the reaction is easily outnumbered by the yield of mutagenesis product, which is double-stranded (ds) due to the modified primer design (see also Figure S2). This allows for the direct transformation of *S. cerevisiae* without the need of prior template removal. The mutagenesis product is then recircularized by homologous recombination of the double-stranded ends. **B,** Analysis of *S. cerevisisae* transformants obtained from the modified SDM protocol shows a high mutagenesis efficiency. Successful mutagenesis in this case results in the addition of an *Bst*BI restriction site into the *BSEP* coding sequence. Colony PCR of the resulting transformants was performed with primers surrounding the mutagenesis site, and the resulting product was digested with *Bst*BI. 19 of the 25 tested clones carried the additional restriction site (+), corresponding to a mutagenesis efficiency of 76%.

Classic SDM relies on the removal of non-mutated template plasmid achieved by *Dpn*I digestion, which recognizes and cleaves only methylated DNA template. Thereby only the mutated plasmid can give colonies. Plasmids prepared directly from *S. cerevisiae*, however are, unmethylated and the template plasmid cannot be removed by *Dpn*I digestion [19], [20], [21]. Thereby, the efficiency of mutagenesis is too low. To obtain positive clones, several hundred nanograms of mutated plasmid are needed and a substantial amount of time is required to pick and analyze several clones to find the correctly mutated plasmid [22], [23], [24]. By changing the mutagenesis primer design from a complete to a partial, 5′-overlap of the primer pair (Figure 2A, step 1), the linear *in vitro* mutagenesis step is turned into an exponential polymerase chain reaction: due to this primer shift a product is generated which carries priming sites that serve as a template in the subsequent reaction cycles (step 2). The usefulness of such a primer shift was previously reported, although in a different context [25]. The reaction product is therefore endowed with homologous double-stranded ends that allow the precise recirculation by homologous recombination into an intact plasmid (step 3; for a detailed comparison of both, classic and DREAM mutagenesis, see Figure S2). A change of primers to mutated plasmid DNA used for transformation thereby increases the probability of picking positive mutated clones.

We analyzed the efficiency of this yeast-based mutagenesis method by introducing a mutation into *BSEP* on a *S. cerevisiae* plasmid that resulted in an additional recognition site for the restriction enzyme *Bst*BI (Figure 2B). The mutagenesis reaction gave ∼100 colonies after transformation. Twenty-five of the resulting transformants were picked and the mutated region of the plasmid was amplified by colony PCR. The product of 1240 bp was then subjected to *Bst*BI digestion, which - assuming that the site directed mutagenesis has been successful - should result in two bands of 960 and 280 bp, respectively. Since no *Bst*B1 site was present in the amplified DNA, the PCR product of colonies not bearing the mutation cannot be digested by this restriction enzyme. From 25 clones picked, 19 carried the introduced mutation, corresponding to a mutagenesis efficiency of 76%. Five of these mutated plasmids where sequenced and all of them were positive for the mutation. The efficiency of two other mutations generated by this strategy was 74%. This demonstrates that our method is comparable to the 80% efficiency reported for the *E. coli*-dependent, classical SDM [26].

### Rapid DREAM mutagenesis and expression analysis of a yeast-enabled mammalian BSEP expression vector

**Figure 3:**
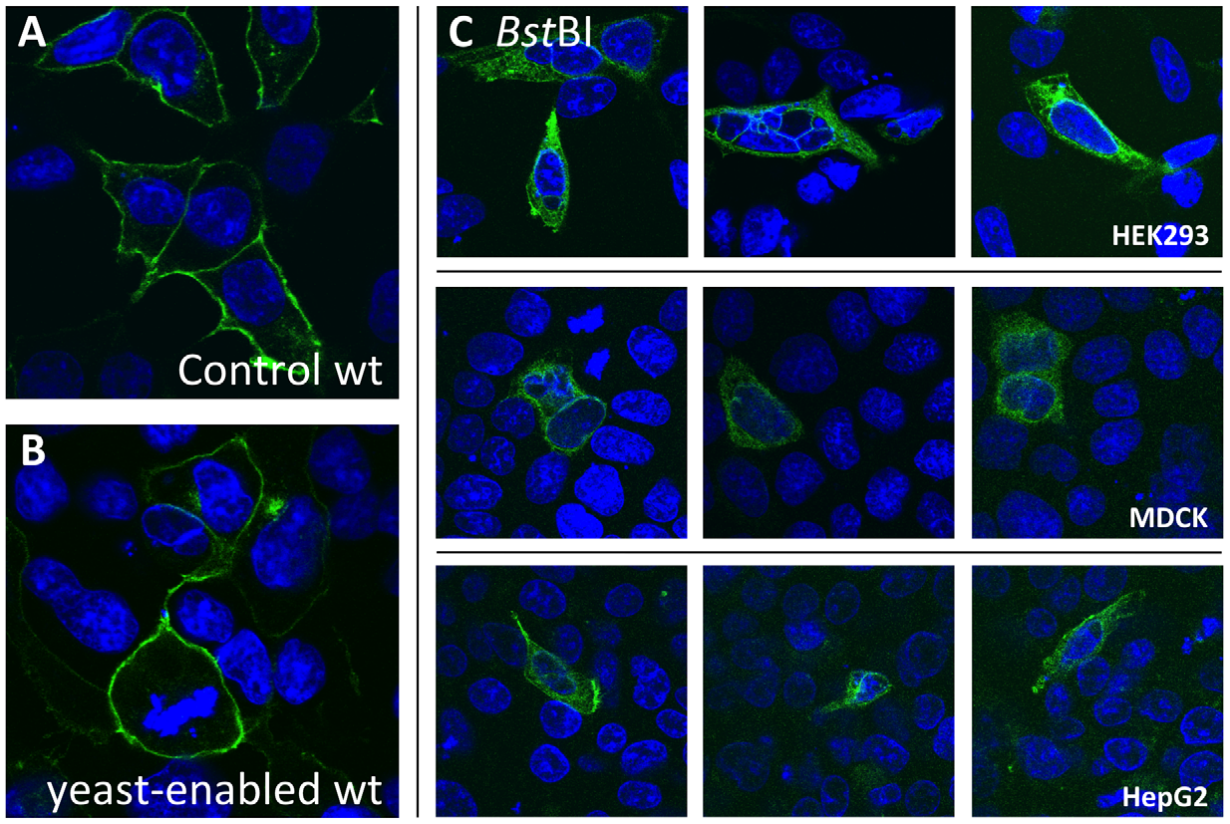
The yeast-maintainable *BSEP* expression plasmid is functionally indistinguishable from the non-yeast parental plasmid in mammalian cell culture. **A,** HEK293 cells were transfected with pEYFP-N1-*BSEP*. **B,** HEK293 cells were transfected with pEYFP-N1-OriLeu-*BSEP*. Equimolar amounts of both constructs showed comparable transfection efficiencies and expression levels (established by FACS analysis, see Figure S3). **C,** HEK293 (upper panel), MDCK (middle), and HepG2 cells (lower panel) were transfected with the *Bst*BI mutation in pEYFP-N1-OriLeu-*BSEP* that was introduced with the *E. coli*-free site-directed mutagenesis method. All cells were transfected with equimolar amounts of the respective constructs via Lipofectamine according to the manufacturer's guidelines. After fixation, nuclei were stained with Hoechst 34580 (blue), and the fluorescence (green) of the YFP tag in the BSEP fusion protein was observed.

Many eukaryotic membrane proteins are expressed and studied in mammalian cell lines [27], [28]. In order to extend the usage of the DREAM method to human cell culture, a yeast-compatible derivative of the mammalian *BSEP* expression vector pEYFP-N1-*BSEP* [29] (Figure S1) was created by introducing the necessary *S. cerevisiae* ORI and selection marker(in analogy to the *P. pastoris* vector modification shown in Figure 1A). After introduction and sequence verification, the obtained plasmid construct was successfully transfected into HEK293 cells as visualized by detection of the fluorescent YFP tag at the C-terminus of the BSEP fusion protein (Figure 3). Comparison with the parental non-yeast expression vector showed that in both cases BSEP is localized at the plasma membrane (Figure 3A and B). Additionally, flow cytometric analyses of HEK293 cells transfected with equimolar amounts of BSEP in pEYFP-N1 and in pEYFP-N1-OriLeu, respectively, were performed to determine transfection and expression rates of both plasmids (Figure S3). Here, transfection rates and BSEP expression from the larger yeast shuttle vector and the smaller, parental vector were found to be comparable (21% and 32%, respectively). These experiments, show that the presence of the additional ORI and selection marker does not influence the expression of BSEP in mammalian cell cultures.

We introduced a *Bst*BI mutation by the *E. coli*-free DREAM method as described above. After plasmid recovery, expression was analyzed by transfection in different mammalian cell lines (Figure 3C). The green fluorescence of the BSEP mutant fusion protein could be detected in HEK293, MDCK and HepG2 cells. Here, a mislocalization of the mutated ABC transporter from the plasma membrane to intracellular compartments could be observed (Figure 3C). This mislocalization has also been observed in a liver biopsy from a patient carrying this *BSEP* mutation (Kubitz and Häussinger, unpublished data).

## Discussion

Here we present a complete, yeast-based workflow to create and manipulate unstable or toxic genes for any expression system. By using this method, we were able to express the “toxic” human wild-type *BSEP* cDNA both in *S. cerevisiae* and *P. pastoris*. Furthermore it is possible to rapidly generate and study BSEP mutations and to use the resultant plasmids in yeast and mammalian expression systems without further modifications. We describe and provide proof of principle for DREAM, a new site-directed mutagenesis method that allows the site-specific manipulation of plasmids in *S. cerevisiae*. This method can be used for many plasmids by simple addition of the sequence necessary for *S. cerevisiae* plasmid maintenance. Moreover, these plasmids can also be obtained in sufficient quantity and quality directly from *S. cerevisiae* cultures [30]. The presented approach can rely on *S. cerevisiae* as the single organism used in all cloning steps from plasmid construction and propagation as well as its mutagenesis.

Homologous recombination has other additional advantages: since the recombination process is completely independent of restriction sites, the in-frame fusion of an insert to e.g. a plasmid-encoded tag never results in unwanted additional amino acids on the recombinant protein, while this is often the case in restriction- or LIC-based cloning [31], [32] (ligation-independent cloning). At the same time, homologous recombination is generally as high-throughput-compatible as LIC.

### DREAM - Directed REcombination-Assisted Mutagenesis

The modified primer design of the yeast-based DREAM mutagenesis method is simple and like the classic SDM covers all aspects of mutagenesis: mutation, insertion and deletion (see Figure S2). The DREAM mutagenesis primer pair can easily be designed by the following basic rules: each oligonucleotide should be around 50 bases long, with 20 bases of 5′ primer-to-primer overlap for an efficient recombination of the mutated plasmid ends in yeast, carrying the mutation, deletion or insertion in their middle, and 30 bases of 3′-sequence for template annealing. The 20 bp overlap on both ends of the linear mutagenesis product results in efficient homology-based gap repair in *S. cerevisiae* to form the circular, mutated plasmid (Figure 2). To minimize the occurrence of errors, the number of thermal cycles is restricted to 18 as recommended for the conventional SDM kit [26]. Accordingly, all analyzed DREAM clones so far replicated the template plasmid sequence i.e. apart from the introduced mutation the whole *BSEP* coding sequence was found to be unchanged.

The new mutagenesis method permits the rapid realization of patient-derived *BSEP* mutations for immediate study in cell cultures. Using DREAM, we could show that a BSEP mutation identified in a patient with progressive familial intrahepatic cholestasis type 2 results in a trafficking defect of the mutant protein that prevents BSEP from being correctly incorporated into the plasma membrane. Future mutations can be generated quickly for their study in mammalian cell lines and/or *in vitro* on the isolated recombinant protein. This is a major advantage since the realization of for example BSEP mutations was previously a work-intensive and time-consuming task. Glycosylation has been shown to be irrelevant for the function of other human ABC transporters expressed in yeast in the past [12], [33]. Furthermore, BSEP expressed in Sf9 cells, which also harbors a glycosylation pattern different from the human pattern, was functional [2]. Thus, it is very likely that the glycosylation state and/or pattern of BSEP is not relevant for its function.

### Concluding remarks

We have established and validated a complete new workflow for the cloning and manipulation of “unstable or toxic” DNA. We believe that in particular the new *E. coli*-independent DREAM mutagenesis strategy will be helpful for proteins where functionally relevant mutants of proteins could not be studied due to limitations of their bacterial cloning.

## Materials and Methods

### Yeast strains and growth conditions

The *Saccharomyces cerevisiae* strain used in this study was the S288C derivative YRE1001 (MATa; *ura3–52*; *trp1-1*; *leu2–3,112*; *his3–11, 15*; *ade2-1*; *pdr1–3*;Δ*PDR5*,Δ*PDR5prom::TRP1*) [34]. For expression in *Pichia pastoris*, strain GS-115 from Invitrogen was used. Yeast cells were maintained either on YPD agar (1% (w/v) yeast extract, 2% (w/v) peptone from caseine, 2% (w/v) glucose, and 2% (w/v) agar for solid media) or synthetic complete (SC) minus leucine media [35] at 30°C, and liquid cultures were shaken at 200 (*S. cerevisiae*) or 250 rpm (*P. pastoris*), respectively.

### Molecular biology reagents, kits, and oligonucleotides

All polymerase-based reactions were performed with the Phusion ® High Fidelity DNA polymerase (NEB) and the supplied HF buffer. Restriction enzymes were from NEB and Fermentas. Oligonucleotides were ordered from Eurofins MWG Operon (Ebersberg, Germany). Plasmid miniprep kits were obtained from Qiagen (Hilden, Germany), and the Nucleobond ® Xtra midiprep kit from Macherey-Nagel was used (Düren, Germany).

### Generation of PCR products and preparation of vectors for homologous recombination in *S. cerevisiae*

The Clone Manager Suite 6 (Sci-Ed Software) was used to design all oligonucleotides and calculate annealing temperatures of the primer pairs. All oligonucleotides and plasmids used in this study can be found in Table S1 and S2, respectively. All PCR reactions were performed with the Phusion® DNA polymerase in HF buffer according to the recommendations of the manufacturer. 50 µl reactions contained 1.5 µl of DMSO. PCR conditions were: 2 min initial denaturation, 50 sec cycle denaturation, 50 sec annealing, 20 sec per kbp of extension for 35 cycles, followed by 7 min of final extension. For homologous recombination, all vectors were linearized by restriction digest as indicated during which they also were dephosporylated with calf intestinal alkaline phosphatase (Fermentas). YEpMDR1HIS was double-digested with *Bam*HI and *Bsm*I, YEpHIS was cut with *Bam*HI and *Mlu*I to remove the C-terminal his tag [12]. The oligonucleotides YEpNHISFor and YEpNHISRev encoding an N-terminal his_14_ tag followed by a factor X_a_ cleavage site were mixed in equimolar amounts, heated to 95°C for 5 min and allowed to anneal by slow cooling to room temperature. After T4 polynucleotide kinase (NEB) treatment, the phosphorylated synthetic insert was ligated into the gel-purified linearized YEpHIS plasmid. *BSEP* PCR products with fitting overlaps were generated with the primer pairs *BSEP*-YEpHISN-S1/-S2 and *BSEP*-YEpHISC-S1/-S2, respectively.

### Transformation of competent *S. cerevisiae* cells

Competent cells were generated as described elsewhere [24]. Briefly, a 5 ml overnight culture was used to inoculate 50 ml YPD to an OD_600_ of 0.2. Cells were harvested at an OD_600_ of 0.8 to 1 and washed once with 50 ml of sterile water. The cell pellet was then washed once in 1 ml of LATE buffer (0.1 M lithium acetate, 10 mM Tris-HCl pH 8, 1 mM EDTA) and adjusted to 50 µl LATE per 10 OD_600_ equivalents of cells. Cells were directly used or stored for a maximum of two days at 4°C. Competent yeast cells were transformed either with intact plasmid or equimolar amounts of DNA fragments for homologous recombination using the Lithium-Acetate/PEG method [24] without single-stranded DNA, and transformants were selected on solid SC minus leucine media. The DNA for yeast transformation was salt/ethanol-precipitated, briefly dried and directly redissolved in the yeast suspension. Resulting transformants were directly used to inoculate 5 ml overnight cultures. From these, 5 ml cultures were inoculated to an OD_600_ of 0.3 and harvested in the early logarithmic growth stage (OD_600_ of 1).

### Analysis of BSEP expression in *Pichia pastoris*

The primer pair OriLeu-pPIC3.5-*Nde*I-S1/-S2 was used to amplify the region of the YEpHIS plasmid containing the 2 micron ori and the leucine prototrophy marker (the sequence of YEpLac181, the parental vector of YEpHIS1 containing the ori and marker information can be found under Genbank acc. no. X75460.1), and *BSEP* was amplified with primers *BSEP*-pPIC3.5-S1/-S2. pPIC3.5 was double-digested with *Nde*I and *Bam*HI, and all fragments were pooled in equimolar amounts and used for transformation of *S. cerevisiae*. After plasmid recovery in *E. coli* XL1blue, the *Pichia pastoris* strain GS-115 was transformed according to the Invitrogen guidelines. Clones obtained from transformation were subjected to a second round of selection on MD plates without histidine. From these, 10 ml MGY cultures were grown overnight at 30°C and 250 rpm shaking. Cells were pelleted at an OD_600_ between 2 to 6 (1500 g, 5 min, 4°C) and resuspended in MMY medium to a final OD_600_ of 1. After additional growth for 24 h, 10 OD_600_ equivalents were harvested and used to make whole cell lysates. Cells were washed once with water, resuspended in 1 ml of water, and lysed on ice for 10 min by addition of 150 µl of YEX buffer (1.5 M NaOH, 7.5% (v/v) β-mercaptoethanol [36]). The solution was precipitated by addition of 150 µl of 50% (w/v) trichloroacetic acid for 10 min on ice, and the precipitate was harvested by centrifugation (10 min, 14.000 rpm, 4°C). After complete removal of supernatants, the pellets were dried briefly at room temperature and resuspended in 100 µl of sample buffer (for composition, see [36]). Samples were incubated for 10 min at 65°C and spun down briefly. 0.5 OD_600_ equivalents were then loaded onto 7% SDS gels and separated at 150 V. After semi-dry electroblotting (Biorad) onto nitrocellulose, the membrane was blocked for 30 min in TBS-T with 5% non-fat dried milk and then probed with a 1∶2500 dilution of the polyclonal rabbit antiserum K168 directed against human BSEP [28].

For *S. cerevisiae*, 2 OD_600_ equivalents of cells were taken and processed. the pellets were resuspended in 80 µl of sample buffer, and 20 µl (0.5 OD_600_ equivalents) were resolved on a 7% SDS gel.

### The *E. coli*-free site-directed mutagenesis (DREAM)

For introduction of the missense, *Bst*BI mutation into *BSEP* constructs, the mutagenesis primer pair *BSEP-Bst*BImut-S1/-S2 was used. Cycling conditions were as in the classic SDM protocol, with 18 cycles to avoid PCR-induced errors, and with the annealing temperature strictly being kept at 60°C to ensure the generation of ds-ended mutagenesis product (also see Figure S1). Reactions were set up with the Phusion ® DNA polymerase, as this proofreading enzyme has a low error rate practically identical to the enzyme used in the Stratagene SDM kits (according to the manufacturers' datasheets: 4.4×10^−7^ for Phusion ® High Fidelity Polymerase, 4.3×10^−7^ for PfuUltra ® HF Polymerase). Extension time was 1 min per kb to allow for complete extension. The recommended elongation time for Phusion polymerase is 15–30 seconds per kbp, and an extended incubation at the elongation step was found to be important for a successful exponential product generation, probably because it ensures the quantitative integrity of the ends that serve as priming sites in subsequent cycles. 10 ng of template was used, and the reaction was initiated after heating the reaction to 98°C (hot start) with 0.5 µl of Phusion polymerase. The reaction was precipitated with sodium acetate and ethanol, and directly resuspended in 20 µl ( = 4 OD_600_ equivalents) of fresh competent yeast cells in LATE buffer. This assures an optimal ratio of mutagenesis product to yeast cells. The transformation was carried out as described above by addition of 120 µl PLATE buffer (40% (v/v) PEG 4000, 0.1 M lithium acetate, 10 mM Tris-HCl pH 8, 1 mM EDTA).

### Yeast colony PCR

Freshly growing transformant colonies were picked and restreaked onto selective agar plates. The remaining of the material was treated with Zymolyase for 45 min at 37°C in 50 µl reaction volume (0.1 M sodium phosphate buffer pH 7.4, 2 mM DTT, and 5–10 mg/ml of Zymolyase T-100 (ICN)). After heating the reactions to 95°C for 10 min, the material was frozen at −20°C for 10 min and thawed again. 5 µl of this were used as template for colony PCR (30 µl reaction in Phusion HF buffer: 2 mM final conc. of MgCl_2_, 20 pmol/primer, 200 µM dNTPs, 1.5 units Phusion ® DNA polymerase). 10 µl of the reaction was directly digested with *Bst*BI (15 µl reaction, 8 units restriction enzyme) for 1 h at 37°C and then resolved on a 1% agarose gel.

### Plasmid recovery from yeast

Small- (5–10 ml for *E. coli* transformation) or large-scale (0.5–1 l for obtaining preparative plasmid amounts) overnight yeast liquid yeast cultures were harvested, washed in cold water once, and then resuspended in Zymolyase incubation buffer (see above, with 1.2 M sorbitol). The yeast cell wall was digested for 30 min at 37°C, cells were then lysed by alkaline lysis as described in [30]. 0.5–1 µl of this preparation was used to transform chemically competent *E. coli* strain XL1blue. After heat shock, bacteria were shaken for 1 h at 30°C and 200 rpm. This low temperature is crucial to prevent the loss of unstable construct. After plating out on low salt LB media containing 50 µg/ml Carbenicillin, plates were incubated at 30°C. Carbenicillin allows for a tighter and longer lasting selection as it hydrolyzes much slower than ampicillin. 150 ml low salt LB with Carbenicillin were then directly inoculated with a single colony and allowed to grow for 36 to 48 hours at 30°C and 200 rpm. Cells were harvested and plasmid prepared from these. The plasmids were sequence-verified.

### Transfection of cell lines with wt and BstBI-mutated BSEP-YFP and immunofluorescence analyses

pEYFP-N1-*BSEP* was made yeast-compatible as described for pPIC3.5 by addition of an Ori/Leu PCR product made from YEpHIS with primer pair OriLeu-pEYFP-*Afl*II-S1/-S2.

HepG2 cells (ATCC, ordering number HB-8065) were cultured in Dulbecco's modified Eagle's medium Nutrimix F12 (DMEM-F12; Invitrogen), HEK293 cells (ATCC, ordering number CRL-1573) were cultured in DMEM and MDCK cells were cultured in MEM with Earle's Salts, each containing 10% fetal calf serum (PAA, Coelbe, Germany), in a humidified, 5% CO_2_-atmosphere at 37°C. The indicated *BSEP*-YFP plasmid DNA was transfected using FuGENE ® HD (Roche) according to the manufacturer's guidelines. For fluorescence microscopy (LSM 510, Zeiss, Oberkochen, Germany) cells were fixed and permeabilized with methanol (100%, 4°C, 1 min) and nuclei were stained with Hoechst 34580 (Invitrogen).

### Flow cytometric analysis

HEK293 cells transiently transfected with equimolar amounts of *BSEP*wt in pEYFP-N1 (1 µg) and in pEYFP-N1-OriLeu (1.38 µg), respectively, and untransfected cells were cultured in 12-well culture plates until subconfluence. For flow cytometric analyses they were washed with ice-cold phosphate buffered saline (PBS) and incubated with Accutase at 37°C. Cells were transferred into 1.5 ml tubes, centrifuged for 30 sec at 4500× g and resuspended in FACS buffer (PBS+5% (v/v) FCS+0,1 % (w/v) NaN_3_). Cell size, granularity and fluorescence intensities were measured by a FACSCanto Flow Cytometer (Becton Dickinson, Heidelberg, Germany) with excitation at 488 nm. EYFP fluorescence was measured at 530±30 nm. Transfected cells were gated by comparison with the fluorescence of untransfected control cells.

## Supporting Information

Figure S1 **Maps of the “yeast-enabled” plasmids used in this study for expression and DREAM mutagenesis of the unstable** ***BSEP*** **cDNA.** **A,** pPIC3.5-OriLeu-*CHISBSEP* for heterologous expression in *Pichia pastoris*. **B,** pEYFP-N1-OriLeu-*BSEP* for expression in mammalian cell culture. Plasmid features used for propagation in *E. coli*, *S. cerevisiae*, and the organism used for BSEP expression are indicated in red, green, and blue, respectively.
(DOC)

Figure S2 **A simple modification of the classic site-directed mutagenesis protocol allows the mutagenesis of toxic or unstable plasmids without the need for** ***E. coli*** **.** The classic site-directed mutagenesis (left cartoon side) results in a mutated and linear plasmid with single-stranded 5′-overhangs formed by the mutagenesis primers. Since the primers are absolutely complementary to each other, the product of the SDM reaction exists *de facto* in a non-covalently closed circular form that is nick-repaired after transformation into *E. coli*. The polymerase-involving mutagenesis reaction is, in contrast to standard PCR, non-exponential: the mutagenesis primers completely overlap (step 1), so the only primer binding sites on the generated SDM product would be at its very ends. These, however, are single-stranded (step 2), and cannot, in the second cycle, serve to further amplify the product of the first cycle (step 3). Instead, in each cycle the primers bind to the original template and generate a linear product with themselves forming the single-stranded 5′-overhangs (step 4). Along with the use of a proofreading DNA polymerase, this assures a minimum of PCR-introduced mutations, as only the original plasmid is copied and thus mutated in each of the 18 reaction cycles. However, because of the low product yield resulting from the linear template amplification, it is also necessary to remove the unmutated template to minimize the chance of picking wild type clones after transformation. The restriction enzyme *Dpn*I is generally used to recognize and digest both the methylated plasmid template and hemimethylated heteroduplex strands while leaving intact the unmethylated mutagenesis product. Native yeast DNA, however, is unmethylated. We solved both the problem of low product yield and false positives by changing the mutagenesis primer design from a complete to a partial, 5′-overlap of the pair (right cartoon side, step 1). A shift of primer positions has previously been reported in a different context [4] and this changes the mutant strand synthesis reaction into a true exponential PCR reaction, because with this primer design a product is generated (step 2) that carries binding sites for the primers and can serve as template in the subsequent reaction cycles (step 3). In classic SDM, the template has to compete with the oligonucleotides for priming, because they allow for just as much base pairing as itself, if the primer-template mismatch due to the mutation is not taken into account (left cartoon side, step 1). In contrast, the reduction of the primer complementarity to 20 bases greatly favors the 30 plus 20 bases annealing to the template in the first cycle (right cartoon side, step 2). More importantly yet, it still outcompetes in subsequent cycles, when the primers can anneal to the plasmid template with 50 bases and to the product template with 30 bases as compared to the 20 bases of unproductive primer-primer annealing. While the 10 additional bases do not drastically favor mutagenesis product priming in the second cycle, already at the end of this cycle the first product molecules with double-stranded ends appear, which subsequently are drastically preferred priming targets offering the full 50 bases. To make sure that the ends of the final reaction product are double-stranded, the annealing temperature is strictly kept at 60°C, which is well above the melting temperature for a 20-mer and prevents the protection of single-stranded ends by unproductive primer annealing.
(DOC)

Figure S3 **FACS analysis of the unmodified and “yeast-enabled” mammalian BSEP expression vector pEYFP-N1-** ***BSEP*** **.** The data indicate that while pEYFP-N1-OriLeu is transfected at a somewhat reduced yet comparable efficiency as the unmodified construct, while the mean fluorescence is even slightly higher. Taken together with Figure 3, this clearly shows that the addition of the Ori/Leu segment to the vector backbone does not compromise construct performance. The data shown here is representative for three independent transfection experiments.
(DOC)

Table S1 **PCR primers used in this study.**
(DOC)

Table S2 **Plasmids used in this study.**
(DOC)

## References

[1] Byrne JA, Strautnieks SS, Mieli-Vergani G, Higgins CF, Linton KJ (2002). The human bile salt export pump: characterization of substrate specificity and identification of inhibitors.. Gastroenterology.

[2] Noe J, Stieger B, Meier PJ (2002). Functional expression of the canalicular bile salt export pump of human liver.. Gastroenterology.

[3] Byrne JA, Strautnieks SS, Ihrke G, Pagani F, Knisely AS (2009). Missense mutations and single nucleotide polymorphisms in ABCB11 impair bile salt export pump processing and function or disrupt pre-messenger RNA splicing.. Hepatology.

[4] Vu K, Bautos J, Hong M-P, Gelli A (2009). The functional expression of toxic genes: Lessons learned from molecular cloning of CCH1, a high-affinity Ca2+ channel.. Anal Biochem.

[5] Ma H, Kunes S, Schatz PJ, Botstein D (1987). Plasmid construction by homologous recombination in yeast.. Gene.

[6] Gibson DG (2009). Synthesis of DNA fragments in yeast by one-step assembly of overlapping oligonucleotides.. Nucleic Acids Res.

[7] Oldenburg KR, Vo KT, Michaelis S, Paddon C (1997). Recombination-mediated PCR-directed plasmid construction in vivo in yeast.. Nucleic Acids Res.

[8] Stieger B, Meier Y, Meier PJ (2007). The bile salt export pump.. Pflugers Arch.

[9] Strautnieks SS, Bull LN, Knisely AS, Kocoshis SA, Dahl N (1998). A gene encoding a liver-specific ABC transporter is mutated in progressive familial intrahepatic cholestasis.. Nat Genet.

[10] Evans GL, Ni B, Hrycyna CA, Chen D, Ambudkar SV (1995). Heterologous expression systems for P-glycoprotein: E. coli, yeast, and baculovirus.. J Bioenerg Biomembr.

[11] Kuchler K, Thorner J (1992). Functional expression of human mdr1 in the yeast Saccharomyces cerevisiae.. Proc Natl Acad Sci USA.

[12] Figler RA, Omote H, Nakamoto RK, Al-Shawi MK (2000). Use of chemical chaperones in the yeast Saccharomyces cerevisiae to enhance heterologous membrane protein expression: high-yield expression and purification of human P-glycoprotein.. Arch Biochem Biophys.

[13] Urbatsch IL, Wilke-Mounts S, Gimi K, Senior AE (2001). Purification and characterization of N-glycosylation mutant mouse and human P-glycoproteins expressed in Pichia pastoris cells.. Arch Biochem Biophys.

[14] Wang Z, Stalcup LD, Harvey BJ, Weber J, Chloupkova M (2006). Purification and ATP Hydrolysis of the Putative Cholesterol Transporters ABCG5 and ABCG8†.. Biochemistry.

[15] Chloupková M, Pickert A, Lee J-Y, Souza S, Trinh YT (2007). Expression of 25 human ABC transporters in the yeast Pichia pastoris and characterization of the purified ABCC3 ATPase activity.. Biochemistry.

[16] Dean M, Rzhetsky A, Allikmets R (2001). The human ATP-binding cassette (ABC) transporter superfamily.. Genome Res.

[17] Stenson PD, Mort M, Ball EV, Howells K, Phillips AD (2009). The Human Gene Mutation Database: 2008 update.. Genome Med.

[18] Braman J, Papworth C, Greener A (1996). Site-directed mutagenesis using double-stranded plasmid DNA templates.. Methods Mol Biol.

[19] Feher Z, Kiss A, Venetianer P (1983). Expression of a bacterial modification methylase gene in yeast.. Nature.

[20] Proffitt JH, Davie JR, Swinton D, Hattman S (1984). 5-Methylcytosine is not detectable in Saccharomyces cerevisiae DNA.. Mol Cell Biol.

[21] Hattman S, Kenny C, Berger L, Pratt K (1978). Comparative study of DNA methylation in three unicellular eucaryotes.. J Bacteriol.

[22] Gietz RD, Woods RA (2001). Genetic transformation of yeast.. Bio Techniques.

[23] Ito H, Fukuda Y, Murata K, Kimura A (1983). Transformation of intact yeast cells treated with alkali cations.. J Bacteriol.

[24] Schiestl RH, Gietz RD (1989). High efficiency transformation of intact yeast cells using single stranded nucleic acids as a carrier.. Curr Genet.

[25] Liu H, Naismith JH (2008). An efficient one-step site-directed deletion, insertion, single and multiple-site plasmid mutagenesis protocol.. BMC Biotechnol.

[26] QuikChange XL. Site-Directed Mutagenesis Kit Instruction Manual.

[27] Tate CG (2001). Overexpression of mammalian integral membrane proteins for structural studies.. FEBS Letters.

[28] Keitel V, Burdelski M, Warskulat U, Kühlkamp T, Keppler D (2005). Expression and localization of hepatobiliary transport proteins in progressive familial intrahepatic cholestasis.. Hepatology.

[29] Keitel V, Burdelski M, Vojnisek Z, Schmitt L, Häussinger D (2009). De novo bile salt transporter antibodies as a possible cause of recurrent graft failure after liver transplantation: A novel mechanism of cholestasis.. Hepatology.

[30] Singh MV, Weil PA (2002). A method for plasmid purification directly from yeast.. Anal Biochem.

[31] Aslanidis C, de Jong PJ (1990). Ligation-independent cloning of PCR products (LIC-PCR).. Nucleic Acids Res.

[32] Geertsma ER, Poolman B (2007). High-throughput cloning and expression in recalcitrant bacteria.. Nat Methods.

[33] Lee SH, Altenberg GA (2003). Expression of functional multidrug-resistance protein 1 in Saccharomyces cerevisiae: effects of N- and C-terminal affinity tags.. Biochem Biophys Res Commun.

[34] Ernst R, Kueppers P, Klein CM, Schwarzmueller T, Kuchler K (2008). A mutation of the H-loop selectively affects rhodamine transport by the yeast multidrug ABC transporter Pdr5.. Proc Natl Acad Sci USA.

[35] Kaiser C, Michaelis S, Mitchell A (1994). Methods in yeast genetics - a laboratory course manual.

[36] Mamnun YM, Schüller C, Kuchler K (2004). Expression regulation of the yeast PDR5 ATP-binding cassette (ABC) transporter suggests a role in cellular detoxification during the exponential growth phase.. FEBS Lett.

