# Supplementary figures and images for: Heterologous Overexpression and Mutagenesis of the Human Bile Salt Export Pump (ABCB11) Using DREAM (Directed REcombination-Assisted Mutagenesis)
Source: PLoS One. 2011 May 31;6(5):e20562. doi: 10.1371/journal.pone.0020562 (PMC3105083; doi:10.1371/journal.pone.0020562)

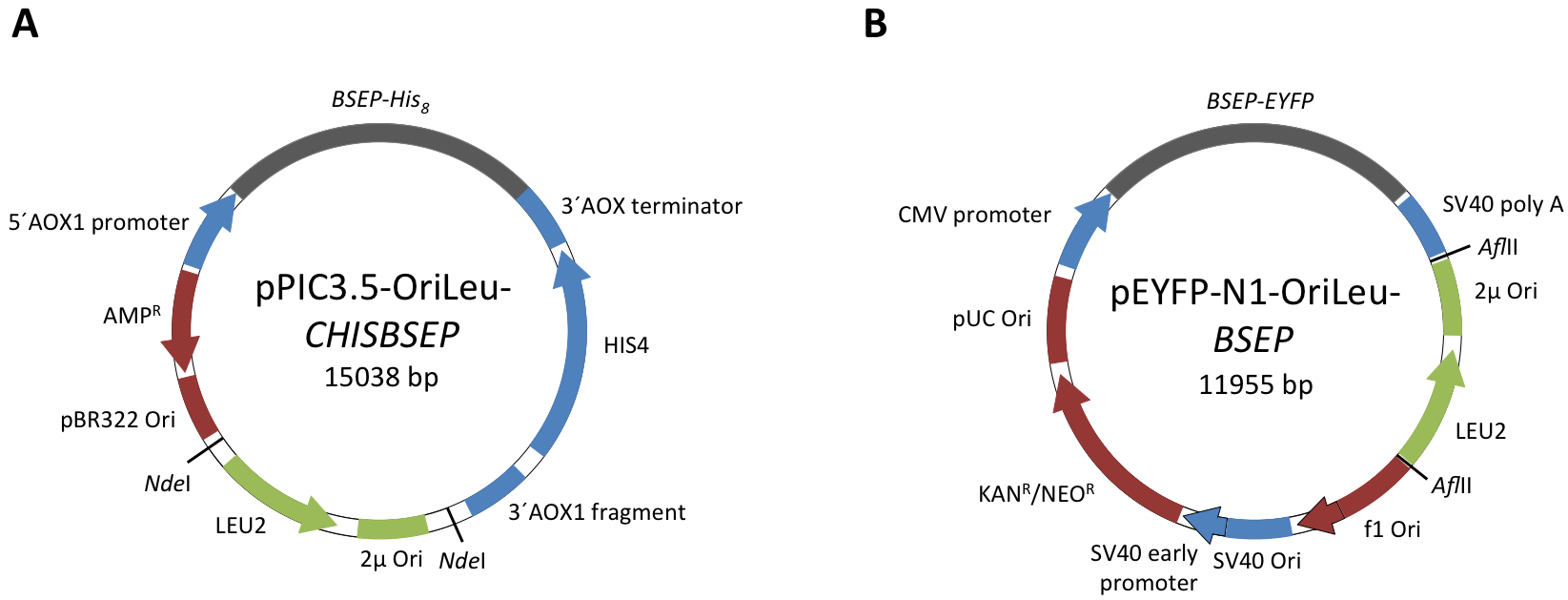

Supplement: Figure S1 — Maps of the “yeast-enabled” plasmids used in this study for expression and DREAM mutagenesis of the unstable BSEP cDNA. A, pPIC3.5-OriLeu-CHISBSEP for heterologous expression in Pichia pastoris. B, pEYFP-N1-OriLeu-BSEP for expression in mammalian cell culture. Plasmid features used for propagation in E. coli, S. cerevisiae, and the organism used for BSEP expression are indicated in red, green, and blue, respectively. (DOC) [file pone.0020562.s001.doc]

**
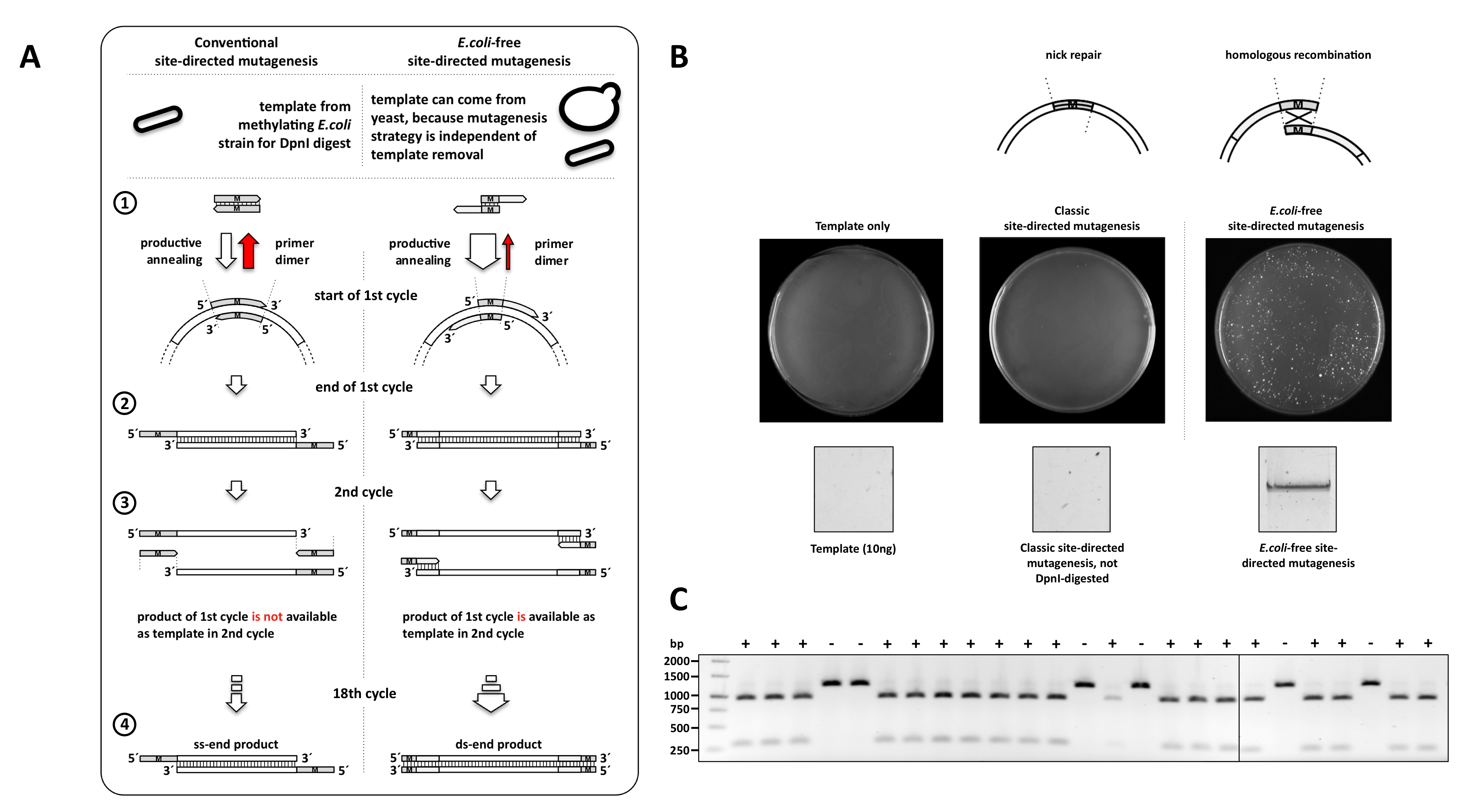
**

Supplement: Figure S2 — A simple modification of the classic site-directed mutagenesis protocol allows the mutagenesis of toxic or unstable plasmids without the need for E. coli . The classic site-directed mutagenesis (left cartoon side) results in a mutated and linear plasmid with single-stranded 5′-overhangs formed by the mutagenesis primers. Since the primers are absolutely complementary to each other, the product of the SDM reaction exists de facto in a non-covalently closed circular form that is nick-repaired after transformation into E. coli. The polymerase-involving mutagenesis reaction is, in contrast to standard PCR, non-exponential: the mutagenesis primers completely overlap (step 1), so the only primer binding sites on the generated SDM product would be at its very ends. These, however, are single-stranded (step 2), and cannot, in the second cycle, serve to further amplify the product of the first cycle (step 3). Instead, in each cycle the primers bind to the original template and generate a linear product with themselves forming the single-stranded 5′-overhangs (step 4). Along with the use of a proofreading DNA polymerase, this assures a minimum of PCR-introduced mutations, as only the original plasmid is copied and thus mutated in each of the 18 reaction cycles. However, because of the low product yield resulting from the linear template amplification, it is also necessary to remove the unmutated template to minimize the chance of picking wild type clones after transformation. The restriction enzyme DpnI is generally used to recognize and digest both the methylated plasmid template and hemimethylated heteroduplex strands while leaving intact the unmethylated mutagenesis product. Native yeast DNA, however, is unmethylated. We solved both the problem of low product yield and false positives by changing the mutagenesis primer design from a complete to a partial, 5′-overlap of the pair (right cartoon side, step 1). A shift of primer positions has previously been reported in a diff [file pone.0020562.s002.doc]

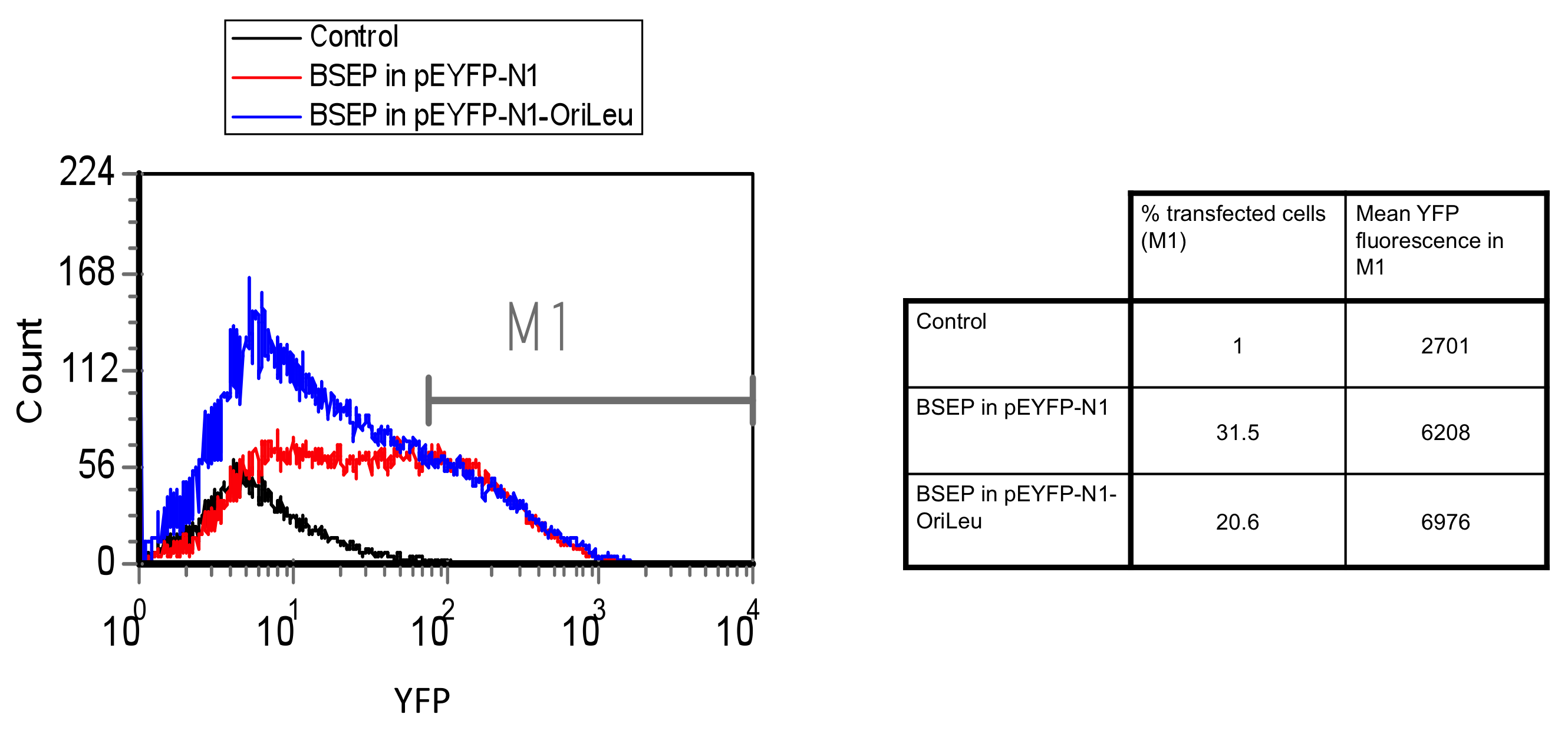

Supplement: Figure S3 — FACS analysis of the unmodified and “yeast-enabled” mammalian BSEP expression vector pEYFP-N1- BSEP . The data indicate that while pEYFP-N1-OriLeu is transfected at a somewhat reduced yet comparable efficiency as the unmodified construct, while the mean fluorescence is even slightly higher. Taken together with Figure 3, this clearly shows that the addition of the Ori/Leu segment to the vector backbone does not compromise construct performance. The data shown here is representative for three independent transfection experiments. (DOC) [file pone.0020562.s003.doc]
