# Supplementary material for: Heterologous Overexpression and Mutagenesis of the Human Bile Salt Export Pump (ABCB11) Using DREAM (Directed REcombination-Assisted Mutagenesis)
Source: PLoS One. 2011 May 31;6(5):e20562. doi: 10.1371/journal.pone.0020562 (PMC3105083; doi:10.1371/journal.pone.0020562)
Supplement: Table S1 — PCR primers used in this study. (DOC) [file pone.0020562.s004.doc]

**Supporting Information S1**

**Table S1. PCR primers used in this study**

| **Oligonucleotide** | **Sequence 5´-3´ (relevant restriction sites in underlined italics and point mutations indicated in bold letter)** | **Used in** |
| --- | --- | --- |
| YEpNHISFor | GATCCTTTAATTATCAAACAATATCAATATGCATCATCACCATCATCACCATCATCACCATCATCATCACCATGGTGGTGGTATTGAAGGTAGACCCGGGTAGA | Supplementary figure 1 |
| YEpNHISRev | CGCGTCTACCCGGGTCTACCTTCAATACCACCACCATGGTGATGATGATGGTGATGATGGTGATGATGGTGATGATGCATATTGATATTGTTTGATAATTAAAG | Supplementary figure 1 |
| *BSEP*-YEpHISN-S1 | TCACCATCATCATCACCATGGTGGTGGTATTGAAGGTAGATCTGACTCAGTAATTCTTCGAAGTATAAAG | Supplementary figure 1 |
| *BSEP*-YEpHISN-S2 | GAATAAGGTAAACATGGTAGCGATGTCGACCTCGAGACGCGTCTAACTGATGGGGGATCCAGTGGTGACT | Supplementary figure 1 |
| *BSEP*-YEpHISC-S1 | ATAAGAAGATAGGATCCTTTAATTATCAAACAATATCAATATGTCTGACTCAGTAATTCTTCGAAGTAT | Supplementary figure 1 |
| *BSEP*-YEpHISC-S2 | CGATGTCGACCTCGAGACGCGTCTAATGGTGATGGTGATGGTGATGGTGACCACTGATGGGGGATCCAGTGGTGACT | Supplementary figure 1 |
| OriLeu-pPIC3.5-*Nde*I-S1 | AACTATGCGGCATCAGAGCAGATTGTACTGAGAGTGCAC*CATATG*CGAGGCCCTTTCGTCTTCAAGAATTAACTGTGGGA | Figure 1 |
| OriLeu- pPIC3.5-*Nde*I-S2 | GTATTTTCTCCTTACGCATCTGTGCGGTATTTCACACCG*CATATG*ATCTGTGCGGTATTTCACACCGCATATATCG | Figure 1 |
| *BSEP*-pPIC3.5-S1 | AATTATTCGAAGGATCCTACGTAGAATTCCCTAGGGCGGCCGCATGTCTGACTCAGTAATTCTTCGAAGTATAAAGAAAT | Figure 1 |
| *BSEP*-pPIC3.5-S2 | TGAGGAACAGTCATGTCTAAGGCGAATTAATTCGCGGCCGCCTAATGGTGATGGTGATGGTGATGGTGACCACTGATG | Figure 1 |
| *BSEP-Bst*BImut-S1 | AGCTC*TT****C****GAA*GAGCCTTCTCTTACACCCCAAGTTATGCAAAAGCTAAA | Figures 2 and 3 |
| *BSEP-Bst*BImut-S2 | AGAAGGCTC*TTC****G****AA*GAGCTGTTGCACTCAGTACAACTGCAGAGATCAC | Figures 2 and 3 |
| OriLeu-pEYFP-*Afl*II-S1 | TCTAGTTGTGGTTTGTCCAAACTCATCAATGTAT*CTTAAG*CGAGGCCCTTTCGTCTTCAAGAATTAACTGTGGGA | Figure 3 |
| OriLeu-pEYFP-*Afl*II-S2 | ATTTTAACAAAATATTAACGCTTACAATTTACGC*CTTAAG*ATCTGTGCGGTATTTCACACCGCATATATCG | Figure 3 |
