# Supplementary material for: Heterologous Overexpression and Mutagenesis of the Human Bile Salt Export Pump (ABCB11) Using DREAM (Directed REcombination-Assisted Mutagenesis)
Source: PLoS One. 2011 May 31;6(5):e20562. doi: 10.1371/journal.pone.0020562 (PMC3105083; doi:10.1371/journal.pone.0020562)
Supplement: Table S2 — Plasmids used in this study. (DOC) [file pone.0020562.s005.doc]

**Table S2. Plasmids used in this study**

| **Plasmid** | **Purpose** | **Reference** |
| --- | --- | --- |
| YEpHIS and YEpMDR1HIS | constitutive; C-terminal his_8_ tag. YEpMDR1HIS contains human *MDR1* cDNA | [12] |
| YEpNHIS | constitutive; N-terminal his_14_ tag | This study |
| YEpNHIS-*BSEP* | constitutive BSEP expression with N-terminal his_14_ tag | This study |
| YEpHIS-*BSEP* | constitutive BSEP expression with C-terminal his_8_ tag | This study |
| pPIC3.5-OriLeu-*CHISBSEP* | yeast-shuttle, *P. pastoris* integration vector for chromosomal BSEP expression with C-terminal his_8_ tag | This study |
| pEYFP-N1-*BSEP* | mammalian BSEP expression plasmid | [29] |
| pEYFP-N1-OriLeu-*BSEP* | yeast-shuttle, mammalian BSEP expression plasmid | This study |
